# Dispersal of the Japanese Pine Sawyer, *Monochamus alternatus* (Coleoptera: Cerambycidae), in Mainland China as Inferred from Molecular Data and Associations to Indices of Human Activity

**DOI:** 10.1371/journal.pone.0057568

**Published:** 2013-02-28

**Authors:** Shao-ji Hu, Tiao Ning, Da-ying Fu, Robert A. Haack, Zhen Zhang, De-dao Chen, Xue-yu Ma, Hui Ye

**Affiliations:** 1 Laboratory of Biological Invasion and Ecosecurity, Yunnan University, Kunming, China; 2 Yunnan Key Laboratory of International Rivers and Transboundary Eco-security, Yunnan University, Kunming, China; 3 Laboratory for Conservation and Utilization of Bio-resource and Key Laboratory for Microbial Resources of the Ministry of Education, Yunnan University, Kunming, China; 4 Laboratory for Animal Genetic Diversity and Evolution of Higher Education in Yunnan Province, Yunnan University, Kunming, China; 5 State Key Laboratory of Genetic Resources and Evolution, Kunming Institute of Zoology, Chinese Academy of Sciences, Kunming, China; 6 USDA Forest Service, Northern Research Station, East Lansing, Michigan, United States of America; 7 Research Institute of Forest Ecology, Environment and Protection, Chinese Academy of Forestry, Beijing, China; 8 The Key Laboratory of Forest Ecology and Environment, State Forestry Administration, Beijing, China; National Center for Biotechnology Information (NCBI), United States of America

## Abstract

The Japanese pine sawyer, *Monochamus alternatus* Hope (Coleoptera: Cerambycidae), is an important forest pest as well as the principal vector of the pinewood nematode (PWN), *Bursaphelenchus xylophilus* (Steiner et Buhrer), in mainland China. Despite the economic importance of this insect-disease complex, only a few studies are available on the population genetic structure of *M. alternatus* and the relationship between its historic dispersal pattern and various human activities. The aim of the present study was to further explore aspects of human activity on the population genetic structure of *M. alternatus* in mainland China. The molecular data based on the combined mitochondrial *cox1* and *cox2* gene fragments from 140 individuals representing 14 Chinese populations yielded 54 haplotypes. Overall, a historical (natural) expansion that originated from China’s eastern coast to the western interior was revealed by the haplotype network, as well as several recent, long-distant population exchanges. Correlation analysis suggested that regional economic status and proximity to marine ports significantly influenced the population genetic structure of *M. alternatus* as indicated by both the ratio of shared haplotypes and the haplotype diversity, however, the PWN distribution in China was significantly correlated with only the ratio of shared haplotypes. Our results suggested that the modern logistical network (i.e., the transportation system) in China is a key medium by which humans have brought about population exchange of *M. alternatus* in mainland China, likely through inadvertent movement of infested wood packaging material associated with trade, and that this genetic exchange was primarily from the economically well-developed east coast of China, westward, to the less-developed interior. In addition, this study demonstrated the existence of non-local *M. alternatus* in new PWN-infested localities in China, but not all sites with non-local *M. alternatus* were infested with PWN.

## Introduction

The Japanese pine sawyer, *Monochamus alternatus* Hope (Coleoptera: Cerambycidae), is a major pest of coniferous forests, especially pines (*Pinus* spp.), and is also the key vector of the exotic pinewood nematode (PWN), *Bursaphelenchus xylophilus* (Steiner et Buhrer) (Nematoda: Aphelenchoididae), in eastern Asia [1]. In Japan and China, the spread of PWN has caused tragic timber losses in pine forests during the past century [2], [3].

Mainland China is one of the areas most highly impacted by PWN in eastern Asia [4], with over 180 localities infested with PWN since 1982 [5]. Dispersal of *M. alternatus* is believed to be responsible for the spread of the PWN in Asia given that PWN is transmitted to new hosts during maturation feeding and oviposition by nematode-laden *M. alternatus* adults [3], [6]. Therefore, studying the dispersal pattern of *M. alternatus* and possible influencing factors could provide critical clues to the spread of PWN in China. It is important to note that *M. alternatus* is native to China, and in the early report by Chen et al. [7] its range was given as being primarily in southern, eastern, and central China with a few isolated populations in western provinces such as Shaanxi and Tibet. Dispersal of *M. alternatus* can be influenced by multiple factors, among which human-mediated movement of the pest is believed to be a significant component. Like many bark- and wood-boring insects [8], [9], *M. alternatus* can be commonly moved to new locations through inadvertent transport of infested wood, including logs, lumber, and wood packaging material. In the case of *M. alternatus*, the portions of a tree trunk or branch that are most commonly infested are the cambial region between the bark and the sapwood and the outer sapwood [10]–[12]. Given the long life cycle of *M. alternatus* and that some larvae and pupae can survive the milling process, wood products made from infested trees, especially the sapwood portion, could serve as a pathway by which *M. alternatus* is moved within China. Although *M. alternatus* could be transported long distances within China via logs and lumber, we believe that wood packaging material such as pallets and crating that are used in the transportation of commercial products is the most likely pathway for long-distance human-assisted transport of *M. alternatus* across mainland China as well as internationally [8], [13]. Hence, we will focus primarily on the movement of wood packaging material in the present paper. In a recent study in China, railways and waterways were significantly linked to the spread of the PWN in China [14]. Given that the modern logistics network of roads, railways, and inland waterways has been associated with human-mediated spread of PWN in China, it is logical that it was also associated with the dispersal of *M. alternatus* in China.

Nowadays, molecular techniques are frequently used to study the dispersal history of invasive organisms or non-local populations of native organisms. For example, Fu et al. [15] stated that the outbreak of the PWN in Yunnan, in southwestern China, was likely caused by inadvertent introduction of *M. alternatus* and PWN from eastern China. Similarly, molecular techniques have been used to explore the origins of other invasive forest pests such as *Agrilus planipennis* Fairmaire (Coleoptera: Buprestidae) and *Tomicus piniperda* (L.) (Coleoptera: Curculionidae: Scolytinae) in North America [16]–[18]. The present research is aimed at investigating the factors that influence the genetic structure of *M. alternatus* in mainland China, and to elucidate the relationship between the genetic structure of *M. alternatus* and both human activity and PWN dispersal in China. The results of the present research should be helpful in explaining how *M. alternatus* has spread in mainland China, formulating integrated management strategies to control *M. alternatus* and PWN, and providing new insights in the field of invasion ecology.

## Materials and Methods

### Sample Collection and Preparation

**Figure 1:**
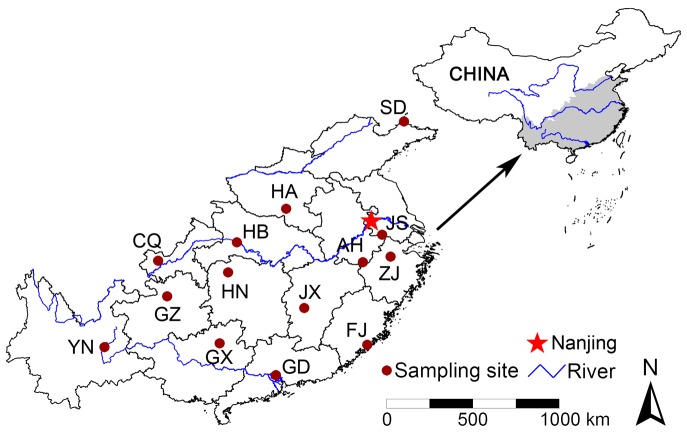
Provincial outline map of our research area in China, indicating the location of the 14 sampling sites as well as the three major rivers in the area (from north to south, Yellow River, Yangtze River, and Pearl River). The two-letter codes correspond to those listed in Table 1. The city Nanjing was marked with a solid star to represent the first reported PWN outbreak area in mainland China.

Fourteen Chinese provinces were selected to cover the major distribution range of *M. alternatus* and PWN in mainland China [5], [7] (Table 1; Fig. 1). Ten individual *M. alternatus* adults were collected from each site with assistance from the local forestry administrations and forest academies using flight-intercept traps (Chinese Academy of Forestry, Zhejiang, China; Fujian Academy of Forestry Sciences, Fujian, China) baited with either the M99-1 bait (Chinese Academy of Forestry) or FJ-Ma-02 bait (Fujian Academy of Forestry Sciences) [19]–[21]. The main attractants in these baits include *α*-pinene, *β*-pinene, and ethanol. The traps were placed in the same pine forests at each location from late March to early October in 2010 and 2011, and were checked weekly for adult beetles.

**Table 1:** Summary information of the 14 sampling localities for *M. alternatus*, arranged by the geographical regions within mainland China*.

Region	Code	Site (province: locality)	Coordinates	Port	PWN	GDP	RFT
Eastern	JS	Jiangsu: Liyang	31°18′ N, 119°27′ E	1	1	2052.93	305.10
	SD	Shandong: Changdao	37°55′ N, 120°44′ E	1	1	2081.32	661.37
	ZJ	Zhejiang: Fuyang	30°02′ N, 119°57′ E	1	1	1470.77	358.48
	FJ	Fujian: Quanzhou	24°54′ N, 118°35′ E	1	1	773.46	172.16
Southern	GD	Guangdong: Guangzhou	23°07′ N, 113°15′ E	1	1	2412.89	396.87
Central	AH	Anhui: Huangshan	29°42′ N, 118°20′ E	0	0	625.58	279.01
	HA	Henan: Queshan	32°49′ N, 113°51′ E	0	1	1196.59	318.00
	HB	Hubei: Yichang	30°52′ N, 110°58′ E	1	1	804.91	168.85
	HN	Hunan: Zhangjiajie	29°07′ N, 110°28′ E	0	0	780.28	175.62
	JX	Jiangxi: Ji’an	27°02′ N, 114°54′ E	0	1	457.92	126.00
Southwestern	CQ	Chongqing: Beibei	29°48′ N, 106°23′ E	1	0	368.08	86.29
	GX	Guangxi: Yongfu	24°58′ N, 109°59′ E	1	0	457.14	137.79
	GZ	Guizhou: Zunyi	27°43′ N, 106°55′ E	0	1	228.62	66.12
	YN	Yunnan: Lunan	24°45′ N, 103°15′ E	0	0	392.04	69.34

* Port indicates whether there was a marine port adjacent to the sampling site, 0 = no, 1 = yes (http://ports.com/browse/asia/china). A trapping site was considered to have an “adjacent” port if the port was located within the same county or city as the collecting site. PWN refers to the historical status of pinewood nematode (PWN) infestation within the same county as where the trapping occurred: 0 = not infested, 1 = infested as of 2012 [5]. GDP values are the 11-year average for the regional gross domestic product (billion yuan) of each of the 14 provinces, NBSC 2001–2011[36]–[46]. RFT values were the 10-year average values of the regional freight turnover (billion tkm) for the 14 sampled provinces, NBSC 2001–2011[36]–[46].

To minimize the chance of sampling closely related individuals, such as individuals from the same parents, 15 ∼ 20 traps were placed 2 ∼ 3 m above ground at a given site and spaced at least 100 m apart. The beetles used from each site were selected from all traps at a location and from both years of sampling. Captured adults were preserved in 95% ethanol and preserved at −40°C until processing in the Laboratory of Biological Invasion and Ecosecurity, Yunnan University. The partial *cox1* and *cox2* sequences of *Monochamus galloprovincialis* (Olivier) from GenBank (EU556596) [22] were selected as the outgroup.

### Ethics Statement

No specific permits were required in the present study for collection of this widespread native forest insect. The authors confirm that the sampling sites were not privately owned or protected. This study did not involve any endangered or protected species.

### Genomic DNA Extraction

Ten *M. alternatus* adults from each of the 14 sampling locations were used in the present research for DNA extraction. All samples were washed with double distilled water and dissected to expose the mesothorax muscle tissues that contain abundant mitochondria. For each individual, 20 mg of muscle tissues were obtained and homogenized in a labeled 1.5 mL Eppendorf tube, and then digested for 12 h with protease buffer, containing 450 *µ*L STE (50 mmol/L NaCl, 100 mmol/L Tris-HCl, and 2 mmol/L EDTA-Na_2_, pH = 8.0; BioBasic Inc., Ontario, Canada), 75 *µ*L SDS (10%; BioBasic Inc.), and 25 *µ*L Proteinase K (20 mg/mL; Merck KGaA, Darmstadt, Germany).

The phenol-chloroform method was applied to the resultant mixture to isolate DNA, and the supernatant was recovered through centrifugation at 5,000×*g*. Next, 550 *µ*L isopropanol was added and chilled at −20°C for 12 h, and then centrifuged at 9,000×*g* to pellet DNA. The pellets were washed with 70% ethanol, dried at 37°C, and dissolved with 60 *µ*L Tris (2 mmol/L; BioBasic Inc.). The dissolved DNA was quantified on an Eppendorf Biophotometer (Eppendorf AG, Hamburg, Germany), and preserved at −40°C in the same laboratory. The ≈100 ng/*µ*L dilutions were used as templates in polymerase chain reactions (PCR).

### PCR Amplification and Sequencing

For all samples, a ≈ 800 bp fragment of the *cox1* gene and a ≈ 700 bp fragment of the *cox2* gene were amplified by PCR on a Biometra T-Professional Standard thermocycler (Biometra GmbH, Göttingen, Germany). The thermal profile consisted of an initial denaturation at 93°C for 3 min; followed by 35 cycles of denaturation at 93°C for 30 sec, annealing at 50°C (*cox1*) or 47°C (*cox2*) for 1 min, and elongation at 72°C for 2 min; then a final elongation at 72°C for 10 min.

The PCR reaction was applied in a 25 *µ*L system which contained 2.6 *µ*L of 10× PCR buffer (Promega, Shanghai, China), 2.6 *µ*L of MgCl_2_ (25 mmol/L; Promega), 4.1 *µ*L of dNTP mixture (2.5 mmol/L each; Fermentas, EU), 0.5 *µ*L of *Taq* DNA polymerase (5 U/*µ*L; Promega), and 0.5 *µ*L of each of forward and reverse primers (10 *µ*mol/L; Sangon Biological Engineering Technology & Services Co. Ltd., Shanghai, China). The primers for the *cox1* fragment were C1-J-2183 (alias “Jerry”) (5′-CAA CAT TTA TTT TGA TTT TTT GG-3′) and TL2-N-3014 (alias “Pat”) (5′-TCC AAT GCA CTA ATC TGC CAT ATT A-3′) [23], and the primers for the *cox2* fragment were tRNA^Leu−F^ (5′-GTG CAA TGG ATT TAA ACC CC-3′) [24] and COII-Croz (5′-CCA CAA ATT TCT GAA CAT TGA CC-3′) [25].

The PCR products were purified using TaKaRa Agarose Gel Purification Kit (version 2.0) (TaKaRa Biotechnology Co., Ltd., Dalian, China) and sequenced by Sangon Biological Engineering Technology & Services Co., Ltd. (Shanghai, China). Sequencing reactions were carried out in both forward and reverse directions on an ABI Prism 3730×l automatic sequencer (Applied Biosystems, Foster City, CA, USA).

### Data Analyses

Raw sequences were proofread and aligned using Clustal W [26] in BioEdit 7.0.9 [27], the product sequences were checked by conceptual translation in MEGA 4.0 [28] to exclude the possibility of obtaining *Numts* (nuclear copies of mtDNA fragments), and then aligned into contigs. The number of polymorphic sites and nucleotide composition were analyzed in MEGA 4.0 [28]. Haplotypes were defined by DAMBE 5.0 [29], [30]. Distances and standard errors between populations were calculated with Kimura’s two-parameter (K2P) model [31] in MEGA 4.0 with 1,000 replications. Nei’s average number of pairwise differences between populations [32], Nei’s average number of pairwise differences within populations, pairwise *F*st values, nucleotide diversity of each population, and the degree of gene flow among populations (*N*_m_) were calculated by Arlequin 3.11 [33]. The significance of Nei’s average number of pairwise differences between populations and the pairwise *F*st values were tested using 1,000 replications.

Transportation (or logistics network) data are difficult to use as a quantified criterion in analysis, but they can be well represented by port distribution and frequency of freight transportation, and are strongly associated with regional economic status [34], [35], therefore, we used port distribution, 11-year average regional freight turnover (RFT, an indicator of transport efficiency, 2001 ∼ 2011) [36]–[46], and 11-year average regional gross domestic product (GDP, 2001 ∼ 2011) [36]–[46] to represent the modern logistics network in mainland China. In an attempt to determine the relationship of these two factors and the current PWN distribution in China to the population genetic structure of *M. alternatus*, an AMOVA (Analysis of Molecular Variance) analysis [47] based on population groups sorted by port distribution (http://ports.com/browse/asia/china), regional economic status [36]–[46], [48], regional freight turnover[36]–[46], and PWN infestation status [5] (Table 1) was performed in Arlequin 3.11. The 14 populations were divided into three economic-status groups based on the ranking in Démurger et al. [48]. Due to the lack of ranking of the RFT data, the 14 populations were divided by different criteria and tested in AMOVA (Table S1), and the combination that resulted in the greatest *F*_CT_ value and the smallest *P* value was considered optimal. The optimal gamma value for AMOVA analysis was estimated using jModelTest 0.1.1 [49], [50].

To analyze the relationship of the above three factors with the population genetic structure of *M. alternatus*, we used each factor in correlation analysis against three key population genetics indices. The first index was the ratio of shared haplotypes (denoted as *R*_shr_) because it can indicate a recent introduction/establishment of a non-local population of a native organism [15], [51], [52]. Two indices like Nei’s average number of pairwise differences within populations (denoted as *K*) and the haplotype diversity (denoted as *D*_hap_) which reflect the genetic diversity within populations were also used in correlation analysis to estimate the influence of human activity on population genetic diversity. Correlation analysis was performed with SPSS 13.0 (SPSS Inc., Chicago, IL, USA), using Kendall’s *τ* and Spearman’s *ρ* methods [53]–[55].

A 3D multidimensional scaling (MDS) analysis [56] based on the K2P (Kimura two-parameter) distances was performed using SPSS 13.0 and a haplotype network was constructed using median joining method in Network 4.6 (Fluxus Technology Ltd., Clare, Suffolk, UK) with the 14 populations categorized by both the geographical regions of mainland China (Table 1) and the indices having significant correlations based on the analyses described above.

The contours of the significantly correlated population genetics indices were mapped in Surfer 10.3 (Golden Software, Inc., Golden, CO, USA) using the Kriging gridding method [57]. The three major rivers in our study area, the distribution of PWN-infested sites as of 2012, and the distribution of coastal and inland marine ports were also mapped for reference. The GIS spatial data of the country-level boundary, provincial boundaries, and rivers of China were obtained from the National Fundamental Geographic Information System (NFGIS) (http://nfgis.nsdi.gov.cn/), while the spatial data for port coordinates were extracted from Ports.com (http://ports.com/browse/asia/china).

## Results

### Sequence Variability and Nucleotide Composition

The alignment yielded a 635 bp *cox1* fragment and a 565 bp *cox2* fragment, which corresponded to the 2,247 ∼ 2,881 bp portion and the 3,079 ∼ 3,643 bp portion in the mitogenome of *Drosophila yakuba* Burla, respectively. In the combined 1,200 bp sequences, 63 sites (5.25%) were polymorphic and 41 sites (3.42%) were informative parsimony. Overall, 40 C–T transitions, 20 A–G transitions, and five A–T transversions were found, consisting of 63.5%, 31.8%, and 7.9% of the total polymorphic sites, respectively. No deletions or insertions were detected. The nucleotide composition showed a high A–T bias of nucleotide usage comprising 73.2% of the total average nucleotide composition.

### Haplotype Distribution

Fifty-four haplotypes of the combined *cox1* and *cox2* sequences were defined based on polymorphic sites and designated in numerical order, with 43 private haplotypes included. Haplotypes derived from the Guangxi and Yunnan populations were locally private, while those derived from Fujian and Zhejiang populations were commonly shared by other populations, with four shared haplotypes present in each population, and the remaining populations containing one to three shared haplotypes (Table 2). The number of private haplotypes also varied among the 14 populations, with six in the Hubei population being the most, while the Henan population had none (Table 2). The *cox1* and *cox2* gene fragments of all the defined haplotypes were separately deposited in GenBank (accession numbers: *cox1*: JX022780 ∼ JX022833, *cox2*: JX022834 ∼ JX022887; some sequences of either gene fragment are identical as the haplotypes were defined by the combined data).

**Table 2:** Haplotype distribution and the number of shared and private haplotypes (underlined) in the 14 populations of *M. alternatus*.

Population	Haplotype(s)	Shared	Private
Jiangsu	H33, H34, H35, H36, H49, H50	2	4
Shandong	H41, H42, H43, H44, H45	3	2
Zhejiang	H47, H48, H49, H50, H51, H52, H53, H54	4	4
Fujian	H8, H9, H10, H13, H32, H45, H48	4	3
Guangdong	H11, H12, H13, H14, H43, H52	3	3
Anhui	H1, H2, H3, H4, H49, H50	2	4
Henan	H50, H52	2	0
Hubei	H19, H20, H21, H22, H23, H24, H25, H29, H32	3	6
Hunan	H26, H27, H28, H29, H30, H31, H32	2	5
Jiangxi	H37, H38, H39, H40, H43, H48	2	4
Chongqing	H5, H6, H7, H19	1	3
Guangxi	H15, H16, H17	0	3
Guizhou	H18, H42	1	1
Yunnan	H46	0	1

### Genetic Variation and Gene Flow

The K2P distances between *M. alternatus* populations varied from 0.0018 to 0.0082, with the distance between the Guizhou and Yunnan populations being smallest while the distance between the Guangxi and Yunnan populations was the largest. Most of the K2P distances between populations from Jiangsu, Shandong, Zhejiang, Fujian, Guangdong, and Anhui were below 0.0040, while the distance between the two populations mentioned above (Guangxi and Yunnan) and those from Henan, Hunan, or Jiangxi were higher. Close genetic connections between geographically distant *M. alternatus* populations were observed, particularly between the populations from Shandong, Henan, or Guizhou and those from Jiangsu, Zhejiang, Fujian, Guangdong, or Anhui (Table S2). Nei’s average number of pairwise differences between *M. alternatus* populations varied from 2.1 to 9.8, with the value between the Guizhou and Yunnan populations being smallest, while the value between the Guangxi and Yunnan populations was the largest. Most of the values between the Jiangsu, Shandong, Zhejiang, Fujian, Guangdong, Anhui, and Hunan populations were below 5.0 and insignificant (Table S3). Overall, the K2P distances and Nei’s average number of pairwise differences showed similar trends in the genetic relationships between populations.

The pairwise *F*st values varied from −0.0026 to 0.0974, with the values between Anhui and Jiangsu populations being smallest, while those between Henan and Yunnan populations were the largest. All pairwise *F*st values between populations from either Guizhou or Yunnan and the remaining populations were above 0.05 and significant at the 0.01 level. However, most pairwise *F*st values between Jiangsu, Shandong, Zhejiang, Fujian, Guangdong, Anhui, and Hunan populations and the value between the Hubei and Chongqing populations were below 0.1 and insignificant (Table S3). The nucleotide diversity of the 14 populations ranged from 0.00000 to 0.00515, with the Yunnan population having the lowest diversity, while the Hubei population had the highest diversity (Table S3).

The gene flow between populations from Jiangsu and Anhui and between populations from Jiangsu and Zhejiang approached infinity, while the gene flow between the remaining populations varied from 0.0134 to 25.5357. Relatively large gene flow values were detected between populations from Hubei and Chongqing (5.2012), Zhejiang and Hunan (6.5625), Jiangsu and Hunan (7.7254), Zhejiang and Fujian (8.5542), Fujian and Anhui (9.8026), Jiangsu and Fujian (13.6057), Shandong and Fujian (18.5227), and Zhejiang and Anhui (25.5357). By contrast, relatively low values were mostly associated with *M. alternatus* populations from less developed or underdeveloped areas of China such as Henan, Chongqing, Guangxi, Guizhou, and Yunnan (Table S2).

### AMOVA Analysis

The AMOVA analysis was significant when the 14 *M. alternatus* populations were divided into three groups based on regional economic status, with 19.9% of variation explained among groups (*F*_CT_ = 0.199, *P* = 0.003), 32.9% among populations within groups (*F*_SC_ = 0.411, *P* = 0.000), and 47.2% within populations (*F*_ST_ = 0.528, *P* = 0.000). The AMOVA analysis was also significant when the 14 populations were divided into three groups based on the following RFT ranking, <100 billion tkm (tonne-kilometre; tkm is a unit of freight transportation), 100 ∼ 200 billion tkm, and >200 billion tkm. The results showed that 20.1% of the variation was explained among groups (*F*_CT_ = 0.201, *P* = 0.005), 32.7% among populations within groups (*F*_SC_ = 0.410, *P* = 0.000), and 47.2% within populations (*F*_ST_ = 0.529, *P* = 0.000). By contrast, when the 14 populations were divided with respect to port and PWN infestation status, the *F*_CT_ values were not significant and the variation among groups was much lower (*F*_CT_ = 0.056, *P* = 0.145 for ports; *F*_CT_ = 0.022, *P* = 0.255 for PWN infestation status) (Table 3; Table S1).

**Table 3:** Summary results for AMOVA analyses of the 14 populations of *M. alternatus* from China that were compared using three grouping criteria (see details in Table 1).

Grouping criteria	Source of variation	*d.f.*	Variance components	% of variation	*F*	*P*
Port distribution	Among groups	1	0.31784Va	5.64	*F*_CT_ = 0.056	0.145
	Among populations within groups	12	2.56276Vb	45.44	*F*_SC_ = 0.482	0.000
	Within populations	126	2.75883Vc	48.92	*F*_ST_ = 0.511	0.000
	Total	139	5.63943	–	–	–
Regional economic status	Among groups	2	1.16196Va	19.87	*F*_CT_ = 0.199	0.003
	Among populations within groups	11	1.92598Vb	32.94	*F*_SC_ = 0.411	0.000
	Within populations	126	2.75883Vc	47.19	*F*_ST_ = 0.528	0.000
	Total	139	5.84677	–	–	–
Regional freight turnover	Among groups	2	1.17718 Va	20.12	*F*_CT_ = 0.201	0.005
	Among populations within groups	11	1.91544 Vb	32.73	*F*_SC_ = 0.410	0.000
	Within populations	126	2.75883 Vc	47.15	*F*_ST_ = 0.529	0.000
	Total	139	5.85146	–	–	–
Pinewood nematode infestationstatus	Among groups	1	0.12418Va	2.24	*F*_CT_ = 0.022	0.255
	Among populations within groups	12	2.66900Vb	48.07	*F*_SC_ = 0.492	0.000
	Within populations	126	2.75883Vc	49.69	*F*_ST_ = 0.503	0.000
	Total	139	5.55202	–	–	–

### Genetic Structure and Influencing Factors

The correlation analysis showed significant positive correlations between the ratio of shared haplotypes (*R*_shr_) and the PWN infestation status (*τ* = 0.615, *P*<0.01; *ρ* = 0.734, *P*<0.01), as well as the RFT (*τ* = 0.478, *P*<0.05; *ρ* = 0.592, *P*<0.05). Correlation analysis also showed significant positive correlations between haplotype diversity (*D*_hap_) and port distribution (*τ* = 0.480, *P*<0.05; *ρ* = 0.558, *P*<0.05), as well as the regional GDP (*τ* = 0.416, *P*<0.05; *ρ* = 0.588, *P*<0.05). However, the ratio of shared haplotypes (*R*_shr_) was not significantly correlated with the port distribution nor the regional GDP, and also the haplotype diversity (*D*_hap_) was not significantly correlated with the PWN infestation status nor the regional RFT. Similarly, no significant correlations were detected between any human-activity related factor and Nei’s average number of pairwise differences within populations (*K*), or between the *K* value and PWN infestation status (Table 4).

**Table 4:** Correlation coefficients between three indices of *M. alternatus* population genetic structure with measures of human activity and current PWN-infestation status.

Method	Factors	Port	PWN	GDP	RFT
Kendall’s *τ*	*R*_shr_	0.080	0.696**	0.384	0.478*
	*K*	0.212	−0.172	−0.055	−0.077
	*D*_hap_	0.480*	0.288	0.416*	0.303
Spearman’s *ρ*	*R*_shr_	0.091	0.790**	0.523	0.592*
	*K*	0.251	−0.203	−0.002	−0.011
	*D*_hap_	0.558*	0.335	0.588*	0.486

* Significant at the 0.05 level (two-tailed);

** Significant at the 0.01 level (two-tailed).

**Figure 2:**
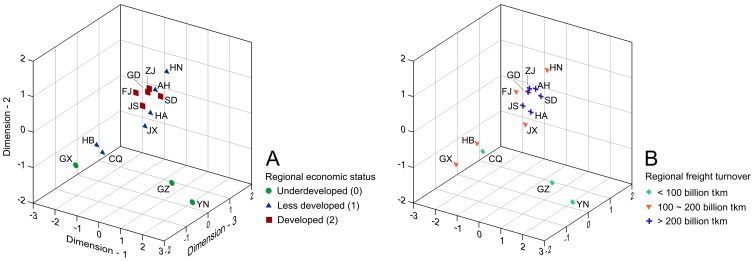
3D Multidimensional scaling (MDS) plots based on Kimura two-parameter (K2P) distances of 14 *M. alternatus* populations in China categorized by regional economic status (A) and regional freight turnover (B). The two-letter population codes in the plots correspond to the provincial codes listed in Table 1.

The 3D MDS analysis categorized the 14 *M. alternatus* populations into three groups (stress = 0.186, RSQ = 0.916). All populations from the economically developed regions and most *M. alternatus* populations from the less developed regions formed one major cluster, while populations from Hubei, Chongqing, and Guangxi formed a second group, and those from Guizhou and Yunnan formed a third group (Fig. 2A). Similarly, populations from regions with denser cargo transportation formed one major cluster, and populations with less cargo transportation formed the other two clusters (Fig. 2B).

**Figure 3:**
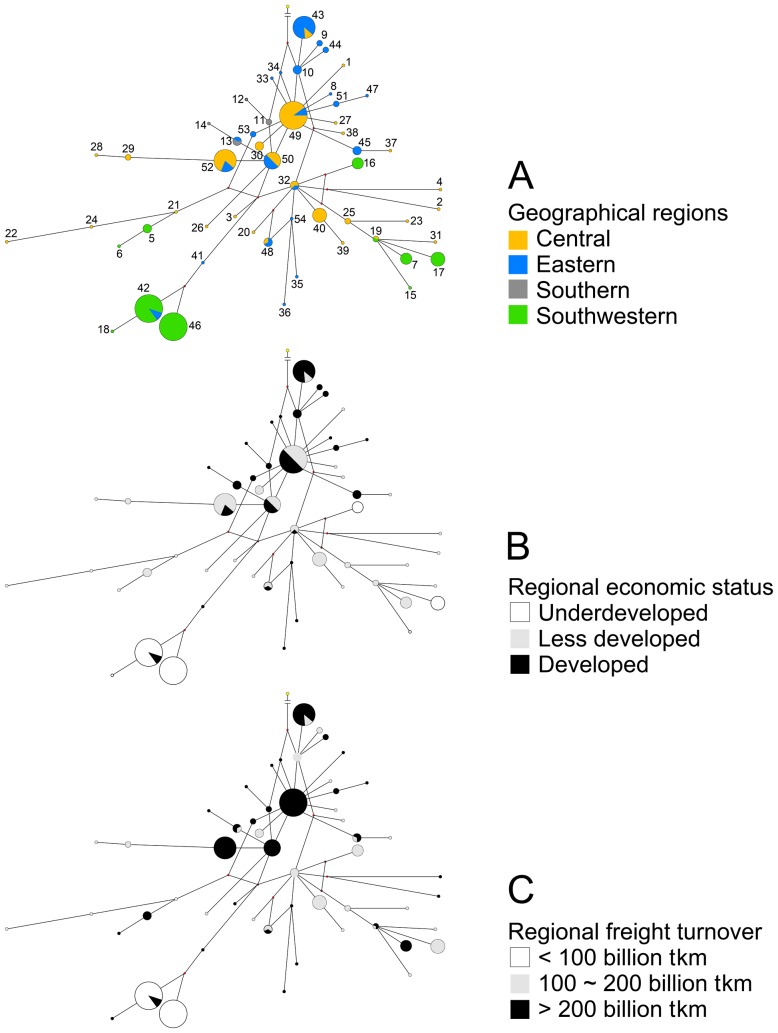
Depicted are the median joining networks of the 54 haplotypes of *M. alternatus* as categorized by (A) the geographical regions of mainland China, (B) the regional economic status, and (C) the regional freight turnover of the 14 provinces within the study region. Node size is proportional to the number of individuals contained in each haplotype; the numbers of haplotypes correspond to the data presented in Table 2.

The median joining network was not clearly structured as evidenced by having at least five loops, nine missing haplotypes (median vectors), and no defined haplogroups. When categorized by the geographical regions of mainland China, the median joining network clearly showed that H10 or H34 from the eastern populations were the ancestry haplotypes based on their direct connection to the outgroup, and a natural (historical) population expansion that occurred from eastern China to central China and then to southern and southwestern China. This network also suggested the existence of human-mediated (recent) dispersal as evidenced by a haplotype shared by geographically distant populations (H42) (Fig. 3A).

When categorized by regional economic status, which was the most significant influencing factor of those analyzed, the median joining network suggested that *M. alternatus* populations expanded from the economically developed regions of China to the less developed regions and then to the underdeveloped regions. Six haplotypes were shared by *M. alternatus* populations from economically developed regions and the less developed regions, while only one haplotype was shared by the populations from the economically developed regions (Shandong) and the underdeveloped regions (Guizhou), and none were shared by populations from the less developed regions and the underdeveloped regions. Twenty-two haplotypes (51.2% of the total number of private haplotypes) were found in populations from the less developed regions, followed by 16 (37.2%) from developed regions, and only five (11.6%) from the underdeveloped regions (Fig. 3B).

**Figure 4:**
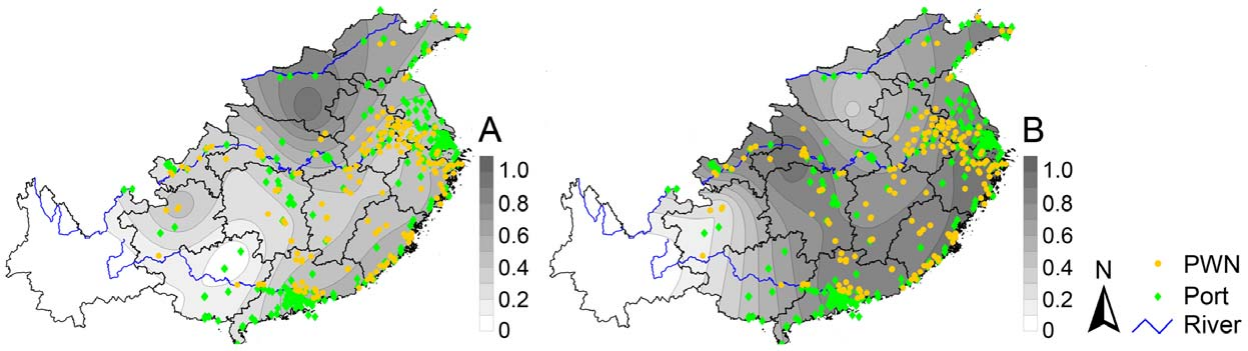
Contour mapping ofthe ratio of shared haplotypes (*R*_shr_) (A) and the haplotype diversity (*D*_hap_) (B) with the distribution of pinewood nematode (PWN) infested sites (yellow dots as of 2012) and ports (green dots) within the research study area in China (as calculated at the provincial level).

The contour mapping of the ratio of shared haplotypes, the haplotype diversity and the distribution of ports and PWN infested sites suggested three relationships. One, sites with PWN infestation were strongly related to port distribution, especially in the deltas of the Yangtze and Pearl Rivers (see river locations in Fig. 1), along the coastal area of China, and along the Yangtze River. Two, areas with higher ratios of shared haplotypes (*R*_shr_) were associated with sites with PWN infestations and ports, especially those along the coastal area and Yangtze River. And three, areas with higher haplotype diversity (*D*_hap_) were also associated with sites with PWN infestations and ports, especially the coastal area and the area stretching from the Pearl River delta upward into central China (Fig. 4).

## Discussion

### Population Genetic Structure

The distribution pattern of *M. alternatus* haplotypes suggests a historical expansion in mainland China from east to west. In the haplotype network (Fig. 3A), the ancestry haplotype (H10 or H34) was defined by several individuals from eastern populations (Jiangsu and Fujian; Table 2), and a dominant haplotype (H49) derived from the ancestry haplotypes was shared by multiple populations in central and eastern China (Jiangsu, Zhejiang, and Anhui; Table 2). Therefore, it is logical to assume that *M. alternatus* populations now present in mainland China first originated in eastern China. The network also suggests that the dominant haplotype subsequently led to the haplotypes now found in coastal China, and the more locally specific haplotypes now found in central or southwestern China.

Our results also indicate genetic proximity between several distantly isolated populations, i.e., Shandong, Zhejiang, Guangdong, Henan, and Guizhou, with shared haplotypes or close K2P distances (Table 2; Fig. 3A; Table S3). These genetic connections reveal that some *M. alternatus* populations in mainland China have recently undergone long-distance exchange. Similar genetic proximity was reported by Kawai et al. and Shoda-Kagaya [24], [58] on the population genetic structure of *M. alternatus* in Japan, as well as in other agricultural pests which are commonly relocated through human activity [52], [59]. The close genetic connections between the above-mentioned distantly-isolated *M. alternatus* populations in the present study also imply human-mediated relocation as reported earlier by Fu et al. [15]. Overall, our results suggest that the genetic structure of *M. alternatus* has been admixed by recent, human-mediated population dispersal of this insect within mainland China.

### Genetic Structure in Association with Human Activities

Although *Monochamus* adults possess the capability to fly several kilometers [60]–[63], long-distance migration over hundreds of kilometers areas is unlikely in nature. In contrast, long-distance spread of many wood-boring insects, such as *M. alternatus*, often results from inadvertent transport of infested wood packaging material [8], and thus it is logical that the recent long-distance spread of *M. alternatus* in China is linked to the national transportation network and the strength of local and regional economies.

In the present study, significant correlations were detected between haplotype diversity (*D*_hap_) and port distribution as well as regional GDP, and between the ratio of shared haplotypes (*R*_shr_) and the RFT (Table 3; Table 4). These relationships are likely linked to the intensity of cargo transportation and its associated wood packaging, which is reflected in a region’s economic status [64], [65], although we acknowledge that *M. alternatus* and PWN can be moved by humans during transport of infested logs and lumber.

In China, the major factor that underpins regional economic status and port distribution is the modern logistics network of highways, rail lines, and waterways that support the massive volumes of cargo that are transported across mainland China [34], [66]. During the past decade, major energy and transportation projects have been promoted in the interior portion of China, especially western China, resulting in a massive flow of industrial equipment from the eastern coastal regions of China [67], [68]. If some of the wood packaging material associated with this cargo was infested with *M. alternatus* then this pest could easily have been transported and introduced to new habitats, resulting in long-distance dispersal with concomitant genetic exchange among populations. Areas with stronger economies often possess denser logistics networks and higher volumes of cargo and associated wood packaging material. Hence, once an area is infested, those areas with well-developed economies would likely have a greater chance of serving as the origin for pests that can easily be moved by human activity, compared to less developed regions. This trend was supported by our 3D MDS plots, as well as the results of the haplotype network, haplotype distribution, genetic distances, pairwise *F*st values, Nei’s average pairwise differences between populations, and gene flow (Figs. 2, 3B, 3C; Table 2; Tables S2, S3). Similarly, the spread of PWN in mainland China has also been linked to transportation networks and population centers in China [14].

**Figure 5:**
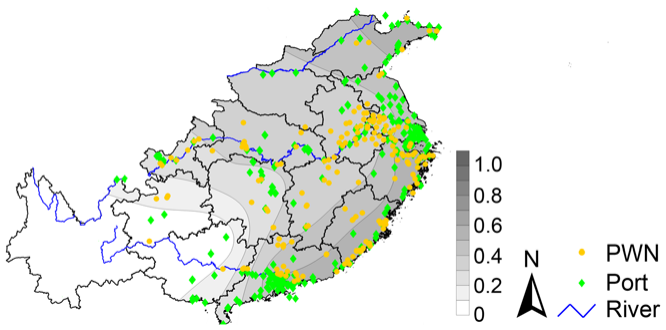
Contour mapping of the ratio of shared *M. alternatus* haplotypes (*R*_shr_) and the distribution of pinewood nematode (PWN) infested sites (yellow dots) and ports (green dots) within our study area in China after exclusion of the *M. alternatus* data from Guizhou and Henan.

Our analyses showed a tendency for dispersal of *M. alternatus* from the coastal, economically developed regions of China, westward to the interior, less developed, and underdeveloped regions of China based on the reductive gradient of the ratio of shared haplotypes (*R*_shr_) (Fig. 4), which could be linked to the intensive transportation of industrial equipment from eastern China to the interior during the past decade. However, the two localities of Henan and Guizhou showed exceptionally higher ratios of shared haplotypes (*R*_shr_) than the neighboring areas (Figure 4A), demonstrating a more recent introduction of non-local *M. alternatus* as reported by the Chinese State Forestry Administration [69], [70]. After isolating these two sites (Henan and Guizhou) from the analysis, the ratio of shared haplotypes (*R*_shr_) in the study area became a reductive gradient from the east coast westward (Fig. 5). Overall, the positive correlation between the genetic structure of *M. alternatus* and regional economic status corroborates that human activity related to commerce and trade is likely a key factor in long-distance spread of *M. alternatus* in China.

### Genetic Structure of *M. alternatus* in Association with PWN Spread

Our analysis showed a significant positive correlation between the distribution of PWN infestations and the ratio of shared *M. alternatus* haplotypes (*R*_shr_) (Table 4), indicating the existence of non-local populations of *M. alternatus* in PWN-infested areas. In nature, spread of the PWN depends on arthropod vectors, with *M. alternatus* being the key vector of PWN in Asia [1], [3], [6], [63], and therefore the spread of PWN is expectedly to be closely linked to the dispersal of *M. alternatus*.

Since the first report of the PWN infestation in China in Nanjing (Fig. 1) in 1982, the PWN-infested area in mainland China has expanded over time, showing a distinct spread pattern from the older infested areas near the Yangtze River Delta, Pearl River Delta, and east coast of China to the newer, more scattered infested localities along the upper Yangtze River and in the western portions of China [5]. Given the genetic evidence for east to west spread of *M. alternatus* in China in the present paper, and the similar spread pattern of PWN in China it can be inferred that westward spread of PWN in mainland China likely resulted from the introduction of non-local *M. alternatus* that originated from areas already infested with PWN.

However, despite the causative role of *M. alternatus* in the spread of the PWN, it is important to realize that not all non-local *M. alternatus* populations carry PWN. This is supported by our finding that there was no significant correlation between the genetic structure and the haplotype diversity (*D*_hap_) in *M. alternatus* with the infestation pattern of PWN (Table 3; Table 4). It is logical that some non-local *M. alternatus* populations can originate from PWN-free areas, or that not all *M. alternatus* from PWN-infested areas carry PWN. Such a scenario was reported in southwestern Yunnan by Fu et al. [15] in which non-local *M. alternatus* were detected in a PWN-free site (Lianghe) while two other nearby sites (Wanding and Ruili) were both infested with PWN. Overall, the present research confirms that the inadvertent introduction of non-local *M. alternatus* can lead to the spread of PWN, but that the invasion and spread of PWN would not necessarily affect the genetic structure of *M. alternatus*.

### Conclusion

Although the present analyses were carried out with a limited sample pool, the composite analyses using molecular and economic data suggested a strong linkage between human activity and the dispersal of *M. alternatus* populations across mainland China in recent years. Advancements in combining molecular biology and socioeconomic indices to address research on the dispersal of cryptic pests will be useful in developing and improving pest management and regulatory strategies, especially when human activities are linked to pest dispersal.

## Supporting Information

Table S1 **Results of AMOVA analyses with significance based on different grouping criteria of regional freight turnover (RFT) in China (see text for details).**
(DOC)

Table S2 **Matrix of the gene flows (** ***N*** **_m_) (above diagonal) and the Kimura two-parameter (K2P) distances (below diagonal) among 14 populations of** ***M. alternatus*** **from mainland China.**
(DOC)

Table S3 **Nei’s average number of pairwise differences between populations (above diagonal), Nei’s average number of pairwise differences within populations (** ***K*** **) (diagonal elements), the pairwise** ***F*** **st values (below diagonal), and the nucleotide diversity (the last row) of the 14 populations of** ***M. alternatus*** **from mainland China.**
(DOC)

## References

[1] Kobayashi F, Yamane A, Ikeda T (1984) The Japanese pine sawyer beetle as the vector of pine wilt disease. Annu Rev Entomol 29: 115–135.

[2] Mamiya Y (1988) History of pine wilt disease in Japan. J Nematol 20: 219–226. 19290205 PMC2618808

[3] Ning T, Fang LY, Tang J, Sun JH (2004) Advances in research on *Bursaphelenchus xylophilus* and its key vector *Monochamus* spp. Entomol Knowledge 41: 97–104. [in Chinese with English abstract].

[4] Wan FH, Guo JY, Zhang F (2009) Research on Biological Invasions in China. Beijing: Science Press: 312.

[5] SFA (State Forestry Administration) (2012) The Bulletin of the State Forestry Administration of P. R. China. (2012-2). Available: http://www.forestry.gov.cn/portal/main/govfile/13/govfile_1894.htm. [in Chinese].

[6] Mamiya Y, Enda N (1972) Transmission of Bursaphelenchus lignicolus (Nematoda: Aphelenchoididae) by Monochamus alternatus (Coleoptera: Cerambycidae). Nematologica 18: 159–162.

[7] Chen S, Xie Y, Deng G (1959) Economic Insect Fauna of China. Fasc. 1. Coleoptera: Cerambycidae - I. Beijing: Science Press: 120. [in Chinese].

[8] Haack RA (2006) Exotic bark- and wood-boring Coleoptera in the United States: recent establishments and interceptions Can J For Res. 36: 269–288.

[9] Haack RA, Hérard F, Sun JH, Turgeon JJ (2010) Managing invasive population of Asian longhorned beetle citrus longhorned beetle: a worldwide perspective. Annu Rev Entomol 55: 521–546. 19743916 10.1146/annurev-ento-112408-085427

[10] Togashi K, Mariga H (1981) Age-specific survival rate and fecundity of the Japanese pine sawyer, *Monochamus alternatus* Hope (Coleoptera: Cerambycidae), at different emergence times. Appl Entomol Zool 16: 351–361.

[11] Togashi K (1989) Development of *Monochamus alternatus* Hope (Coleoptera: Cerambycidae) in relation to oviposition time. Japan J Appl Entomol Zool 33: 1–8.

[12] Wang LP (2004) Study on the biological characteristic of *Monochamus alternatus* Hope. J Fujian Forestry Sci Tech 31: 23–26.

[13] Gu J, Braasch H, Burgermeister W, Zhang J (2006) Records of *Bursaphelenchus* spp. intercepted in imported packaging wood at Ningbo, China. For Pathol 36: 323–333.

[14] Robinet C, Roques A, Pan H, Fang G, Ye J, et al (2009) Role of human-mediated dispersal in the spread of the pinewood nematode in China. PLoS One 4: e4646. 19247498 10.1371/journal.pone.0004646 PMC2645708

[15] Fu DY, Hu SJ, Ye H, Haack RA, Zhou PY (2010) Pine wilt disease in Yunnan, China: evidence of non-local pine sawyer *Monochamus alternatus* (Coleoptera: Cerambycidae) populations revealed by mitochondrial DNA. Insect Sci 17: 439–447.

[16] Carter MCA, Robertson JL, Haack RA, Lawrence RK, Hayes JL (1996) Genetic relatedness of North American populations of *Tomicus piniperda* (Coleoptera: Scolytidae). J Econ Entomol 89: 1345–1353. 8986124 10.1093/jee/89.6.1345

[17] Ritzerow S, Konrad H, Stauffer C (2004) Phylogeography of the Eurasian pine shoot beetle *Tomicus piniperda* (Coleoptera: Scolytidae). Eur J Entomol 101: 13–19.

[18] Bray AM, Bauer LS, Poland TM, Haack RA, Cognato AI, et al (2011) Genetic analysis of emerald ash borer (*Agrilus planipennis*) populations in Asia and North America. Biol Invasions 13: 2869–2887.

[19] Zhao JN, Jiang P, Wu CS, Sun SL, Jiang LY, et al.. (2000) Studies on *Monochamus alternatus* attractant and attractability. For Res 13: 262–267. [in Chinese with English abstract].

[20] Zhao JN, Lin CC, Jiang LY, Wu CS, Yao JF, et al.. (2001) Study on trapping *Monochamus alternatus* and other pine boring beetles with M99-1 liquid attractant. For Res 14: 523–529. [in Chinese with English abstract].

[21] Huang JS, He XY, Yang X, Chen JW, Chen HM (2005) Effect and application of introducing *Monochamus alternatus* in forest with FJ-MA-02 introducing agent. J Fujian Forestry Sci Tech 32: 1–5. [in Chinese with English abstract].

[22] Koutroumpa FA, Lieutier F, Roux-Morabito G (2009) Incorporation of mitochondrial fragments in the nuclear genome (Numts) of the longhorned beetle *Monochamus galloprovincialis* (Coleoptera, Cerambycidae). J Zool Syst Evol Res 47: 141–148.

[23] Simon C, Francesco F, Beckenbach A, Crespi B, Liu H, et al (1994) Evolution, weighting, and phylogenetic utility of mitochondrial gene sequences and a compilation of conserved polymerase chain reaction primers. Ann Entomol Soc Am 87: 651–701.

[24] Kawai M, Shoda-Kagaya E, Maehara T, Zhou ZL, Lian CL, et al (2006) Genetic structure of pine sawyer *Monochamus alternatus* (Coleoptera: Cerambycidae) populations in Northeast Asia: consequences of the spread of pine wilt disease. Environ Entomol 35: 569–579.

[25] Roehrdanz RL (1993) An improved primer for PCR amplification of mitochondrial DNA in a variety of insect species. Insect Mol Biol 2: 89–91. 9087547 10.1111/j.1365-2583.1993.tb00129.x

[26] Thompson JD, Higgins DG, Gibson TJ (1994) CLUSTAL W: improving the sensitivity of progressive multiple sequence alignment through sequence weighting, position-specific gap penalties and weight matrix choice. Nucleic Acids Res 22: 4673–4680. 7984417 10.1093/nar/22.22.4673 PMC308517

[27] Hall TA (1999) BioEdit: a user-friendly biological sequence alignment editor and analysis program for Windows 95/98/NT. Nucleic Acids Symp Ser (Oxf) 41: 95–98.

[28] Tamura K, Dudley J, Nei M, Kumar S (2007) MEGA4: Molecular Evolutionary Genetics Analysis (MEGA) software version 4.0. Mol Biol Evol 24: 1596–1599. 17488738 10.1093/molbev/msm092

[29] Xia XH (2000) Data analysis in molecular biology and evolution. Boston: Kluwer Academic Publishers: 280.

[30] Xia X, Xie Z (2001) DAMBE: software package for data analysis in molecular biology and evolution. J Hered 92: 371–373. 11535656 10.1093/jhered/92.4.371

[31] Kimura M (1980) A simple method for estimating evolutionary rates of base substitutions through comparative studies of nucleotide sequences. J Mol Evol 16: 111–120. 7463489 10.1007/BF01731581

[32] Nei M, Li WH (1979) Mathematical model for studying genetic variation in terms of restriction endonucleases. Proc Natl Acad Sci U S A 76: 5269–5273. 291943 10.1073/pnas.76.10.5269 PMC413122

[33] Excoffier L, Laval G, Schneider S (2005) Arlequin (version 3.0): an integrated software package for population genetics data analysis. Evol Bioinform Online 1: 47–50. PMC2658868 19325852

[34] Jiang B, Prater E (2002) Distribution and logistics development in China: the revolution has begun. Int J Phys Distrib Logist Manag 32: 783–798.

[35] Liang X, Hu CP, Qian K, Mao BH (2011) Study on relations between freight turnover and economic development. Logist Technol 30. [in Chinese with English abstract].

[36] NBSC (National Bureau of Statistics of China) (2001) China Statistical Year Book for Regional Economy 2001. Beijing: China Financial & Economic Publishing House. 603p. [in Chinese with English summary].

[37] NBSC (National Bureau of Statistics of China) (2002) China Statistical Year Book for Regional Economy 2002. Beijing: China Financial & Economic Publishing House. 504p. [in Chinese with English summary].

[38] NBSC (National Bureau of Statistics of China) (2003) China Statistical Year Book for Regional Economy 2003. Beijing: China Financial & Economic Publishing House. 591p. [in Chinese with English summary].

[39] NBSC (National Bureau of Statistics of China) (2004) China Statistical Year Book for Regional Economy 2004. Beijing: China Financial & Economic Publishing House. 620p. [in Chinese with English summary].

[40] NBSC (National Bureau of Statistics of China) (2005) China Statistical Year Book for Regional Economy 2005. Beijing: China Statistics Press. 569p. [in Chinese with English summary].

[41] NBSC (National Bureau of Statistics of China) (2006) China Statistical Year Book for Regional Economy 2006. Beijing: China Statistics Press. 599p. [in Chinese with English summary].

[42] NBSC (National Bureau of Statistics of China) (2007) China Statistical Year Book for Regional Economy 2007. Beijing: China Statistics Press. 581p. [in Chinese with English summary].

[43] NBSC (National Bureau of Statistics of China) (2008) China Statistical Year Book for Regional Economy 2008. Beijing: China Statistics Press. 553p. [in Chinese with English summary].

[44] NBSC (National Bureau of Statistics of China) (2009) China Statistical Year Book for Regional Economy 2009. Beijing: China Statistics Press. 557p. [in Chinese with English summary].

[45] NBSC (National Bureau of Statistics of China) (2010) China Statistical Year Book for Regional Economy 2010. Beijing: China Statistics Press. 567p. [in Chinese with English summary].

[46] NBSC (National Bureau of Statistics of China) (2011) China Statistical Year Book for Regional Economy 2011. Beijing: China Statistics Press. 559p. [in Chinese with English summary].

[47] Excoffier L, Smouse PE, Quattro JM (1992) Analysis of molecular variance inferred from metric distances among DNA haplotypes: application to human mitochondrial DNA restriction data. Genetics 131: 479–491. 1644282 10.1093/genetics/131.2.479 PMC1205020

[48] Démurger S, Sachs JD, Woo WT, Bao SM, Chang G, et al.. (2002) Geography, economic policy, and regional development in China. In: NBER Working Paper Series, No. 8897. Cambridge, MA: National Bureau of Economic Research: 1–62.

[49] Guindon S, Gascuel O (2003) A simple, fast and accurate method to estimate large phylogenies by maximum-likelihood. Syst Biol 53: 696–704. 10.1080/10635150390235520 14530136

[50] Posada D (2008) jModelTest: phylogenetic model averaging. Mol Biol Evol 25: 1253–1256. 18397919 10.1093/molbev/msn083

[51] Nardi F, Carapelli A, Dallai R, Roderick GK, Frati F (2005) Population structure and colonization history of the olive fly, *Bactrocera oleae* (Diptera, Tephritidae). Mol Ecol 14: 2729–2738. 16029474 10.1111/j.1365-294X.2005.02610.x

[52] Shi W, Kerdelhué C, Ye H (2010) Population genetic structure of the oriental fruit fly, *Bactrocera dorsalis* (Hendel) (Diptera: Tephritidae) from Yunnan province (China) and nearby sites across the border. Genetica 138: 377–385. 20012674 10.1007/s10709-009-9429-0

[53] Spearman C (1904) The proof and measurement of association between two things. Am J Psychol 15: 72–101. 3322052

[54] Kendall MG (1938) A new measure of rank correlation. Biometrika 30: 81–89.

[55] Kendall MG (1962) Rank Correlation Methods. London: Charles Griffin & Co, Ltd: 199.

[56] Lessa EP (1990) Multidimensional analysis of geographical genetic structure. Syst Zool 39: 242–252.

[57] Yang CS, Kao SP, Lee FB, Hung PS (2004) Twelve different interplotation methods: a case study of Surfer 8.0. In: Alhan O, editor Proceedings of the XXth ISPRS Congress 35: 778–785.

[58] Shoda-Kagaya E (2007) Genetic differentiation of the pine wilt disease vector *Monochamus alternatus* (Coleoptera: Cerambycidae) over a mountain range - revealed from microsatellite DNA markers. Bull Entomol Res 97: 167–174. 17411479 10.1017/S000748530700483X

[59] Shi W, Kerdelhué C, Ye H (2012) Genetic structure and inferences on potential source areas for *Bactrocera dorsalis* (Hendel) based on mitochondrial and microsatellite markers. PLoS One 7: e37083. 22615898 10.1371/journal.pone.0037083 PMC3353900

[60] Shibata E (1986) Dispersal movement of the adult Japanese pine sawyer, *Monochamus alternatus* Hope (Coleoptera: Cerambycidae) in a young pine forest. Appl Entomol Zool 21: 184–186.

[61] Fujioka H (1993) A report on the habitat of *Monochamus alternatus* Hope in Akita Prefecture. Bulletin of the Akita Prefecture Forest Technical Center 2: 40–56. [in Japanese].

[62] Lai YX (1998) Flight behavior of *Monochamus alternatus* and strategic thoughts to control pine wilt disease. J Zhejiang For Coll 15: 320–323. [in Chinese with English abstract].

[63] Akbulut S, Stamps WT (2012) Insect vectors of the pinewood nematode: a review of the biology and ecology of *Monochamus* species. For Pathol 42: 89–99.

[64] Colunga-Garcia M, Haack RA, Adelaja AO (2009) Freight transportation and the potential for invasions of exotic insects in urban and periurban forests of the United States. J Econ Entomol 102: 237–246. 19253642 10.1603/029.102.0133

[65] Marini L, Haack RA, Rabaglia RJ, Toffolo EP, Battisti A, et al (2011) Exploring associations between international trade and environmental factors with establishment patterns of exotic Scolytinae. Biol Invasions 13: 2275–2288.

[66] Wang CJ (2007) Evolution and developing mechanism of port distribution system in China. Acta Geogr Sinica 62: 809–820. [in Chinese with English abstract].

[67] Cao ZJ, Zhang J, Li J (2012) The history of West Development under the view of industrial transfer. Res Develop 161: 157–161. [in Chinese].

[68] Lan L (2012) Report on the infrastructure construction progress of the western development during the five-year plan 2011–2015. Transpoworld 19: 22–30. [in Chinese].

[69] SFA (State Forestry Administration) (2005) The Bulletin of the State Forestry Administration of P. R. China. (2005-2). Available: http://www.forestry.gov.cn/portal/main/govfile/13/govfile_1280.html. [in Chinese].

[70] SFA (State Forestry Administration) (2011) The Bulletin of the State Forestry Administration of P. R. China. (2011-2). Available: http://www.forestry.gov.cn/portal/main/govfile/13/govfile_1792.htm. [in Chinese].

