# Supplementary material for: Dispersal of the Japanese Pine Sawyer, Monochamus alternatus (Coleoptera: Cerambycidae), in Mainland China as Inferred from Molecular Data and Associations to Indices of Human Activity
Source: PLoS One. 2013 Feb 28;8(2):e57568. doi: 10.1371/journal.pone.0057568 (PMC3585188; doi:10.1371/journal.pone.0057568)
Supplement: Table S1 — Results of AMOVA analyses with significance based on different grouping criteria of regional freight turnover (RFT) in China (see text for details). (DOC) [file pone.0057568.s001.doc]

Table S1: Results of AMOVA analyses with significance based on different grouping criteria of regional freight turnover (RFT) in China (see text for details).

| **Grouping criteria** | **Source of variation** | ***d.f.*** | **Variance components** | **% of variation** | ***F*** | ***P*** |
| --- | --- | --- | --- | --- | --- | --- |
| < 100 tkm  > 100 tkm | Among groups | 1 | 1.90512Va | 28.42 | *F*_CT_ = 0.284 | 0.011 |
|  | Among populations within groups | 12 | 2.03955Vb | 30.43 | *F*_SC_ = 0.425 | 0.000 |
|  | Within populations | 126 | 2.75883Vc | 41.16 | *F*_ST_ = 0.588 | 0.000 |
|  | Total | 139 | 6.70350 | -- | -- | -- |
| < 200 tkm  > 200 tkm | Among groups | 1 | 0.48939Va | 8.56 | *F*_CT_ = 0.086 | 0.048 |
|  | Among populations within groups | 12 | 2.47227Vb | 43.22 | *F*_SC_ = 0.473 | 0.000 |
|  | Within populations | 126 | 2.75883Vc | 48.23 | *F*_ST_ = 0.518 | 0.000 |
|  | Total | 139 | 5.72050 | -- | -- | -- |
| < 100 tkm  100 ~ 200 tkm  > 200 tkm | Among groups | 2 | 1.17718Va | 20.12 | *F*_CT_ = 0.201 | 0.005 |
|  | Among populations within groups | 11 | 1.91544Vb | 32.73 | *F*_SC_ = 0.410 | 0.000 |
|  | Within populations | 126 | 2.75883Vc | 47.15 | *F*_ST_ = 0.529 | 0.000 |
|  | Total | 139 | 5.85146 | -- | -- | -- |
| < 150 tkm  150 ~ 300 tkm  > 300 tkm | Among groups | 2 | 0.70912Va | 12.46 | *F*_CT_ = 0.125 | 0.023 |
|  | Among populations within groups | 11 | 2.22390Vb | 39.07 | *F*_SC_ = 0.446 | 0.000 |
|  | Within populations | 126 | 2.75883Vc | 48.47 | *F*_ST_ = 0.515 | 0.000 |
|  | Total | 139 | 5.69185 | -- | -- | -- |
