# Supplementary material for: Dispersal of the Japanese Pine Sawyer, Monochamus alternatus (Coleoptera: Cerambycidae), in Mainland China as Inferred from Molecular Data and Associations to Indices of Human Activity
Source: PLoS One. 2013 Feb 28;8(2):e57568. doi: 10.1371/journal.pone.0057568 (PMC3585188; doi:10.1371/journal.pone.0057568)
Supplement: Table S2 — Matrix of the gene flows ( N m) (above diagonal) and the Kimura two-parameter (K2P) distances (below diagonal) among 14 populations of M. alternatus from mainland China. (DOC) [file pone.0057568.s002.doc]

Table S2. Matrix of the gene flows (*N*_m_) (above diagonal) and the Kimura two-parameter (K2P) distances (below diagonal) among 14 populations of *M. alternatus* from mainland China.

| **Population** | **AH** | **CQ** | **FJ** | **GD** | **GX** | **GZ** | **HA** | **HB** | **HN** | **JS** | **JX** | **SD** | **YN** | **ZJ** |
| --- | --- | --- | --- | --- | --- | --- | --- | --- | --- | --- | --- | --- | --- | --- |
| Anhui (AH) |  | 0.6663 | 9.8026 | 2.5547 | 0.4700 | 0.2137 | 0.6803 | 1.9107 | 6.3225 | ∞ | 1.2782 | 3.5344 | 0.1453 | 25.5357 |
| Chongqing (CQ) | 0.0059 |  | 0.6173 | 0.5461 | 1.5055 | 0.2402 | 0.3387 | 5.2012 | 1.1678 | 0.6682 | 0.8678 | 0.5491 | 0.1658 | 0.6968 |
| Fujian (FJ) | 0.0029 | 0.0061 |  | 4.6644 | 0.4406 | 0.2002 | 0.6612 | 1.5301 | 3.9634 | 13.6057 | 1.3415 | 18.5227 | 0.1293 | 8.5542 |
| Guangdong (GD) | 0.0031 | 0.0061 | 0.0029 |  | 0.3769 | 0.1773 | 0.9918 | 1.1764 | 3.5227 | 4.3874 | 0.9550 | 3.4375 | 0.1136 | 3.1818 |
| Guangxi (GX) | 0.0060 | 0.0047 | 0.0062 | 0.0064 |  | 0.1607 | 0.2173 | 1.6947 | 0.7807 | 0.4642 | 0.7060 | 0.3958 | 0.1142 | 0.4545 |
| Guizhou (GZ) | 0.0049 | 0.0064 | 0.0051 | 0.0049 | 0.0066 |  | 0.0300 | 0.4076 | 0.3565 | 0.2000 | 0.2619 | 0.2718 | 0.0250 | 0.1970 |
| Henan (HA) | 0.0027 | 0.0053 | 0.0027 | 0.0020 | 0.0055 | 0.0041 |  | 0.7515 | 1.7623 | 0.8206 | 0.4701 | 0.5843 | 0.0134 | 0.8878 |
| Hubei (HB) | 0.0050 | 0.0050 | 0.0053 | 0.0054 | 0.0053 | 0.0060 | 0.0046 |  | 3.8922 | 1.8637 | 2.2153 | 1.1445 | 0.2802 | 1.8852 |
| Hunan (HN) | 0.0039 | 0.0061 | 0.0041 | 0.0040 | 0.0062 | 0.0056 | 0.0031 | 0.0055 |  | 7.7254 | 1.5240 | 2.3972 | 0.2274 | 6.5625 |
| Jiangsu (JS) | 0.0026 | 0.0057 | 0.0027 | 0.0027 | 0.0058 | 0.0047 | 0.0023 | 0.0049 | 0.0037 |  | 1.3636 | 3.0928 | 0.1293 | ∞ |
| Jiangxi (JX) | 0.0044 | 0.0059 | 0.0043 | 0.0045 | 0.0056 | 0.0054 | 0.0039 | 0.0053 | 0.0053 | 0.0041 |  | 1.0285 | 0.1693 | 1.3000 |
| Shandong (SD) | 0.0035 | 0.0070 | 0.0031 | 0.0033 | 0.0072 | 0.0049 | 0.0033 | 0.0061 | 0.0047 | 0.0034 | 0.0051 |  | 0.1758 | 2.5993 |
| Yunnan (YN) | 0.0062 | 0.0080 | 0.0067 | 0.0065 | 0.0082 | 0.0018 | 0.0057 | 0.0072 | 0.0072 | 0.0061 | 0.0070 | 0.0064 |  | 0.1240 |
| Zhejiang (ZJ) | 0.0027 | 0.0056 | 0.0028 | 0.0029 | 0.0059 | 0.0048 | 0.0022 | 0.0049 | 0.0038 | 0.0025 | 0.0042 | 0.0035 | 0.0064 |  |
