# Supplementary material for: Dispersal of the Japanese Pine Sawyer, Monochamus alternatus (Coleoptera: Cerambycidae), in Mainland China as Inferred from Molecular Data and Associations to Indices of Human Activity
Source: PLoS One. 2013 Feb 28;8(2):e57568. doi: 10.1371/journal.pone.0057568 (PMC3585188; doi:10.1371/journal.pone.0057568)
Supplement: Table S3 — Nei’s average number of pairwise differences between populations (above diagonal), Nei’s average number of pairwise differences within populations ( K ) (diagonal elements), the pairwise F st values (below diagonal), and the nucleotide diversity (the last row) of the 14 populations of M. alternatus from mainland China. (DOC) [file pone.0057568.s003.doc]

Table S3. Nei’s average number of pairwise differences between populations (above diagonal), Nei’s average number of pairwise differences within populations (*K*) (diagonal elements), the pairwise *F*st values (below diagonal), and the nucleotide diversity (the last row) of the 14 populations of *M. alternatus* from mainland China.

| **Population** | **AH** | **CQ** | **FJ** | **GD** | **GX** | **GZ** | **HA** | **HB** | **HN** | **JS** | **JX** | **SD** | **YN** | **ZJ** |
| --- | --- | --- | --- | --- | --- | --- | --- | --- | --- | --- | --- | --- | --- | --- |
| Anhui | **3.333** | 7.060** | 3.480 | 3.720** | 7.200** | 5.900** | 3.200** | 6.000** | 4.700 | 3.100 | 5.240** | 4.160* | 7.400** | 3.240 |
| Chongqing | 0.429** | **4.733** | 7.260** | 7.300** | 5.580** | 7.600** | 6.300** | 5.980 | 7.220** | 6.760** | 7.040** | 8.300** | 9.500** | 6.660** |
| Fujian | 0.049 | 0.448** | **3.290** | 3.420 | 7.400** | 6.100** | 3.200** | 6.280** | 4.880* | 3.260 | 5.140** | 3.720 | 8.000** | 3.340 |
| Guangdong | 0.164** | 0.478** | 0.097 | **2.889** | 7.600** | 5.900** | 2.440** | 6.460** | 4.720** | 3.280* | 5.400** | 3.920* | 7.800** | 3.420* |
| Guangxi | 0.515** | 0.249** | 0.532** | 0.570** | **3.644** | 7.900** | 6.600** | 6.360** | 7.400** | 6.900** | 6.700** | 8.600** | 9.800** | 7.000** |
| Guizhou | 0.701** | 0.675** | 0.714** | 0.738** | 0.757** | **0.200** | 4.900** | 7.100** | 6.700** | 5.600** | 6.400** | 5.900** | 2.100** | 5.700** |
| Henan | 0.424** | 0.596** | 0.431** | 0.335** | 0.697** | 0.943** | **0.356** | 5.440** | 3.680** | 2.700** | 4.700** | 4.000** | 6.800** | 2.640** |
| Hubei | 0.207** | 0.088 | 0.246** | 0.298** | 0.228** | 0.551** | 0.400** | **6.178** | 6.520* | 5.820** | 6.360* | 7.280** | 8.600** | 5.820** |
| Hunan | 0.073 | 0.300** | 0.112* | 0.124** | 0.390** | 0.584** | 0.221** | 0.114* | **5.378** | 4.460 | 6.360** | 5.640** | 8.600** | 4.520 |
| Jiangsu | -0.022 | 0.428** | 0.035 | 0.102* | 0.519** | 0.714** | 0.379** | 0.212** | 0.061 | **3.000** | 4.920** | 4.040* | 7.300** | 2.940 |
| Jiangxi | 0.281** | 0.366** | 0.272** | 0.344** | 0.415** | 0.656** | 0.515** | 0.184** | 0.247** | 0.268** | **4.200** | 6.060** | 8.300** | 5.000** |
| Shandong | 0.124* | 0.477** | 0.026 | 0.127* | 0.558** | 0.648** | 0.461** | 0.304** | 0.173** | 0.139 | 0.327** | **3.956** | 7.600** | 4.160** |
| Yunnan | 0.775** | 0.751** | 0.794** | 0.815** | 0.814** | 0.952** | 0.974** | 0.641** | 0.687** | 0.795** | 0.747** | 0.740** | **0.000** | 7.600** |
| Zhejiang | 0.019 | 0.418** | 0.055 | 0.136* | 0.524** | 0.717** | 0.360** | 0.210** | 0.071 | -0.024 | 0.278** | 0.161** | 0.801** | **3.022** |
| Nucleotide diversity | 0.00278 | 0.00394 | 0.00274 | 0.00241 | 0.00304 | 0.00017 | 0.00030 | 0.00515 | 0.00448 | 0.00250 | 0.00350 | 0.00330 | 0.00000 | 0.00252 |

* Significant at the 0.05 level;

** Significant at the 0.01 level.
